# Supplementary material for: Associations Between Daily Outdoor Temperature and Subjective Real-time Ratings of Emotional States and Sleep in Mood Disorder Subtypes
Source: medRxiv. 2025 Sep 9:2025.09.08.25335358. Preprint. [Version 1] doi: 10.1101/2025.09.08.25335358 (PMC12440061; doi:10.1101/2025.09.08.25335358)
Supplement: 1 [file NIHPP2025.09.08.25335358V1-supplement-1.pdf]

SUPPLEMENT

Section 1: Figures

medRxiv preprint doi: https://doi.org/10.1101/2025.09.08.25335358; this version posted September 9, 2025. The copyright holder for this preprint (which was not certified by peer review) is the author/funder, who has granted medRxiv a license to display the preprint in perpetuity.
This article is a US Government work. It is not subject to copyright under 17 USC 105 and is also made available for use under a CC0 license.

Figure S1: Percentage change in Sad Mood with DMOT stratified across seasons and history of mood disorder subtypes.

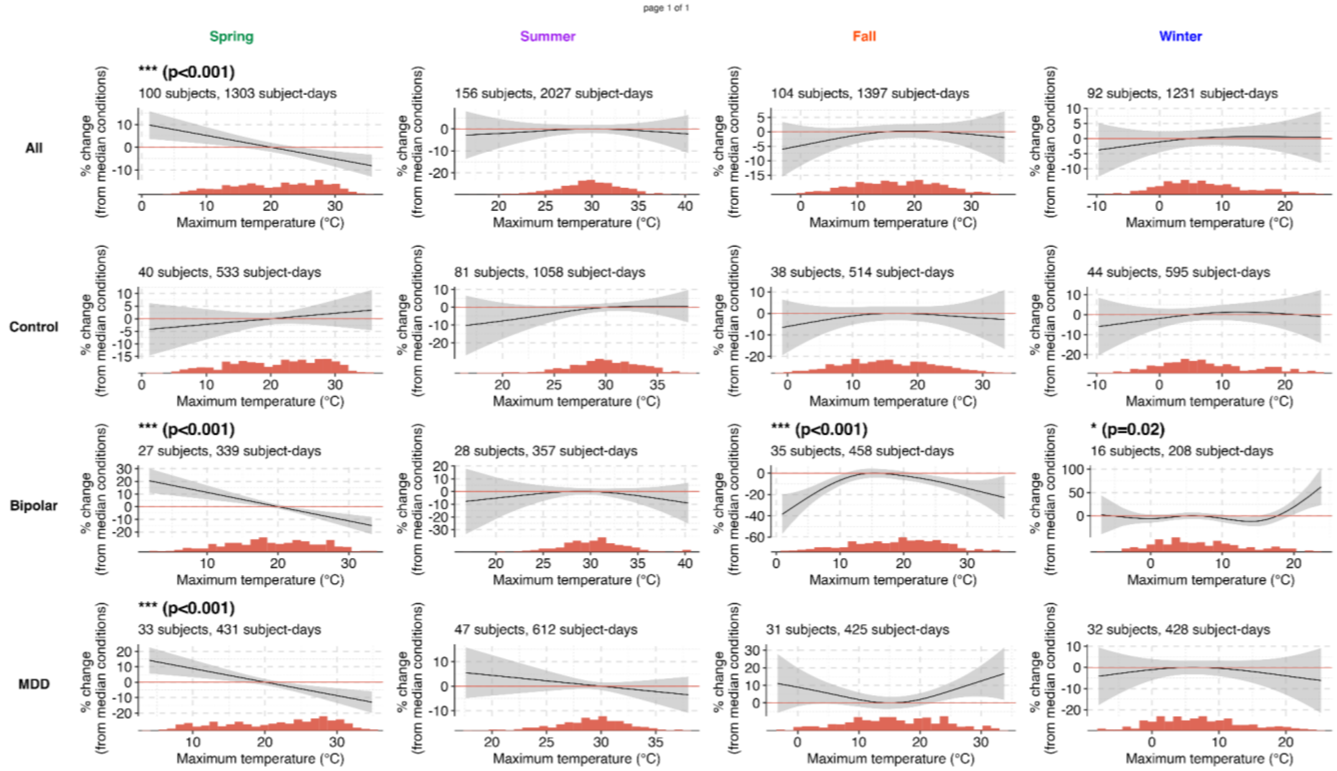

medRxiv preprint doi: https://doi.org/10.1101/2025.09.08.25335358; this version posted September 9, 2025. The copyright holder for this preprint (which was not certified by peer review) is the author/funder, who has granted medRxiv a license to display the preprint in perpetuity.
This article is a US Government work. It is not subject to copyright under 17 USC 105 and is also made available for use under a CC0 license.

Figure S2: Percentage change in ENERGY with DMOT stratified across seasons and history of mood disorder subtypes.

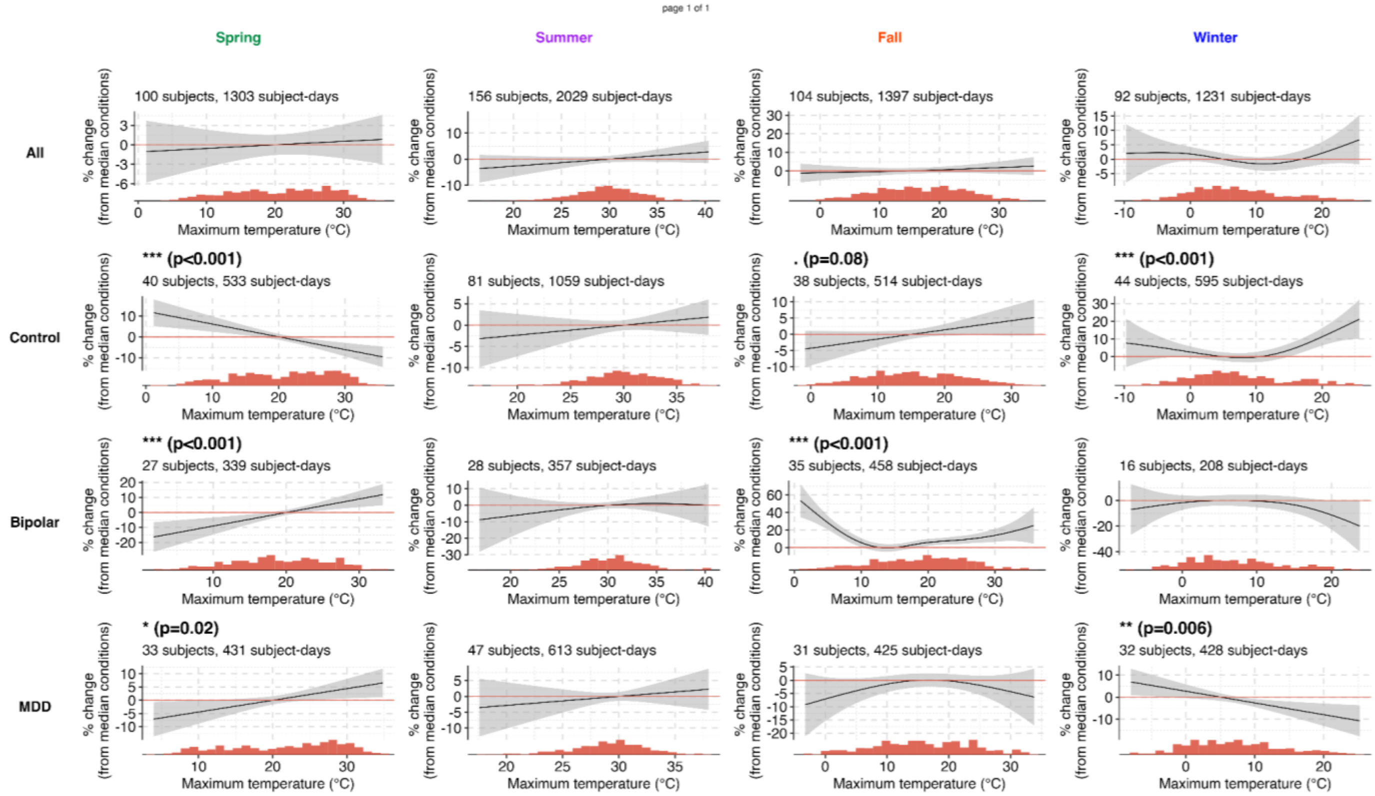

medRxiv preprint doi: https://doi.org/10.1101/2025.09.08.25335358; this version posted September 9, 2025. The copyright holder for this preprint
(which was not certified by peer review) is the author/funder, who has granted medRxiv a license to display the preprint in perpetuity.
This article is a US Government work. It is not subject to copyright under 17 USC 105 and is also made available
for use under a CC0 license.

Figure S3: Percentage change in ANXIOUSNESS with DMOT stratified across seasons and history of mood disorder subtypes.

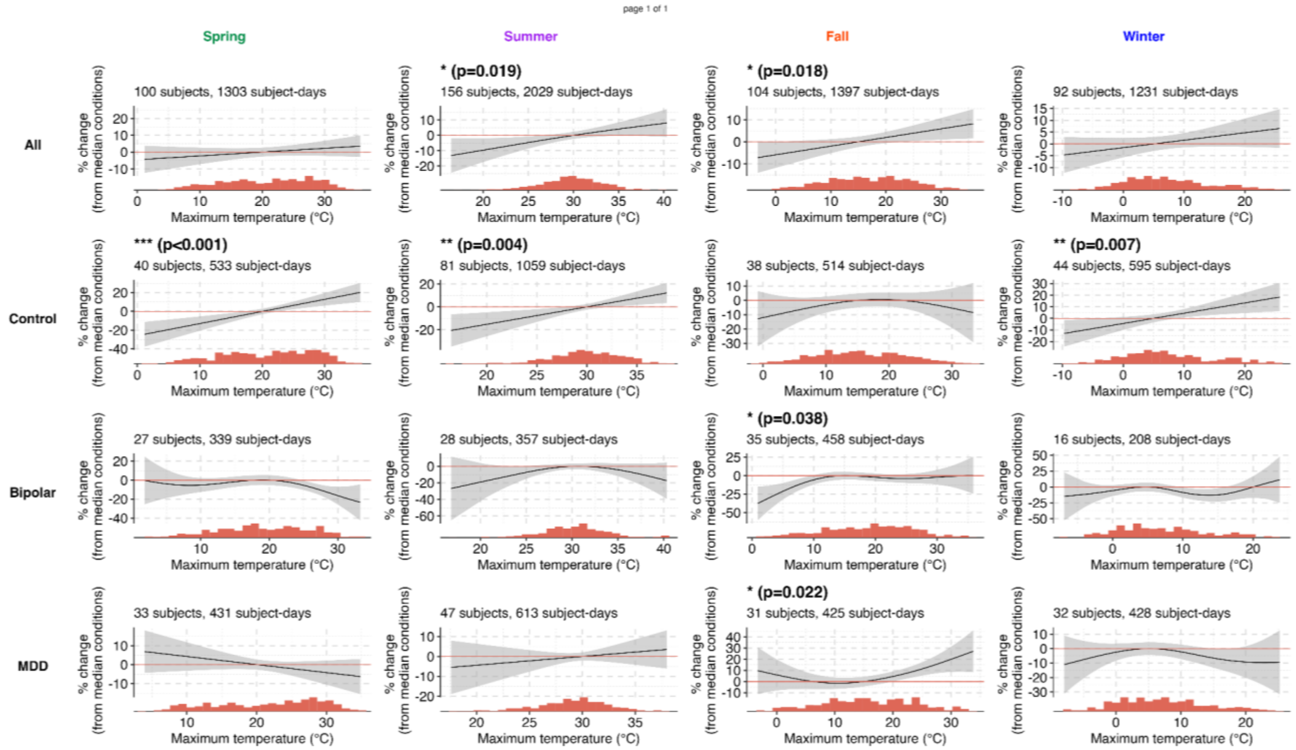

medRxiv preprint doi: https://doi.org/10.1101/2025.09.08.25335358; this version posted September 9, 2025. The copyright holder for this preprint (which was not certified by peer review) is the author/funder, who has granted medRxiv a license to display the preprint in perpetuity.
This article is a US Government work. It is not subject to copyright under 17 USC 105 and is also made available for use under a CC0 license.

Figure S4: Percentage change in SLEEP QUALITY with DMOT stratified by seasons and the history of mood disorder subtypes.

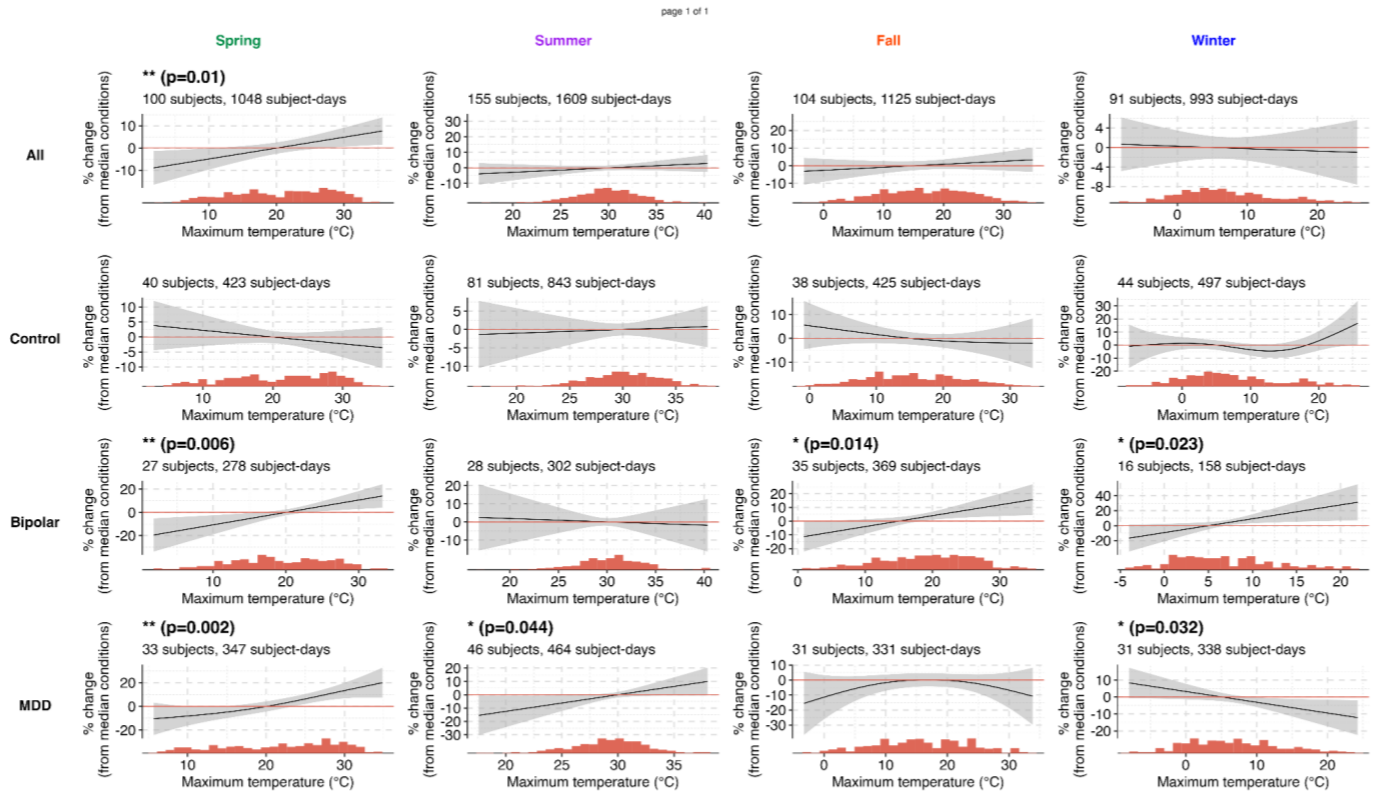

medRxiv preprint doi: https://doi.org/10.1101/2025.09.08.25335358; this version posted September 9, 2025. The copyright holder for this preprint (which was not certified by peer review) is the author/funder, who has granted medRxiv a license to display the preprint in perpetuity.
This article is a US Government work. It is not subject to copyright under 17 USC 105 and is also made available for use under a CC0 license.

SECTION 2: STATISTICAL MODELS

Model set $\boldsymbol{M}^{(s)}$, s = {spring, summer, fall, winter}**:**

$$Y_{ij} = {Z_i}^t\beta + X_{ij}\gamma + \sum_{k=1}^{3} f_k(t_{ij}) * 1\{D_i = k\} + b_i \ ,$$
$$V_{i(j+1)} = {Z_i}^t\beta + X_{ij} \ \gamma + \sum_{k=1}^{3} f_k(t_{ij}) * 1\{D_i = k\} + b_i$$

Model set $\boldsymbol{M}^{(s)}$ denotes the set of models fit on the subsample of subjects from a specific season s. Here, Y_{ij} is the average daily value for an EMA variable (self-reported sad mood/ energy/anxiousness) on day *j* for participant *i* and $V_{i(j+1)}$ is the self-reported sleep quality for $(j+1)$-th day for participants *i*. Z_i and β are the design matrix and regression coefficients associated with non-time-varying adjusters (sex, age, history of mood disorder, and an indicator for whether day *j* is a weekend (Friday/Saturday/Sunday)), b_i is a subject-specific random intercept which accounts for within-subject correlation, X_{ij} is the average daily total cloud cover (in percentage) and t_{ij} is the daily maximum outdoor temperature (DMOT) on day *j* for participant *i*. f_k's denote smooth functions of temperature modeled as a penalized B-spline.. D_i denotes the history of mood disorder for the participant i - where D_i=1 for the **Control** group, D_i=2 for the **Bipolar** group, and D_i=3 for the **MDD** group. The models are fitted using the MGCV package (S. Wood & Wood, 2015) in R Statistical software.
